# Comparison of spirometric parameters in athletes engaged in aerobic and anaerobic sports: a cross-sectional study

**DOI:** 10.1097/MS9.0000000000000729

**Published:** 2023-05-03

**Authors:** Yesha Shree Rajaure, Bikalp Thapa, Lee Budhathoki, Shavana RL Rana, Manoj Khadka

**Affiliations:** Departments of a Physiology; b Community Medicine, Nepalese Army Institute of Health Sciences; c Shree Birendra Hospital, Kathmandu, Nepal

**Keywords:** cross-sectional study, Nepal, spirometry, sports

## Abstract

Pulmonary function tests are better among athletes than non-athletes. The spirometry parameters may vary among athletes involved in aerobic and anaerobic sports. This study aims to study the difference in spirometry parameters among aerobic and anaerobic sports athletes.

**Materials and methods:**

This cross-sectional study was conducted among 131 professional athletes. A convenience sampling technique was used. The data were collected from April to November 2022 using a proforma form, entered into Microsoft Excel, and analyzed using Statistical Package for Social Sciences version 16.

**Results:**

Out of 131 total participants, 65 were involved in aerobic sports, while 66 were in anaerobic sports. The mean age of the participants was 27.34±5 years, the majority being male (*n*=104). Aerobic athletes had higher differences in forced vital capacity (1.19%, *P*=0.726) and forced expiratory volume in the first second (3.08%, *P*=0.315), the difference was not statistically significant. Statistically significantly higher difference in forced expiratory flow at 25–75% (13.42%, *P*=0.001), forced expiratory flow at 0.2–1.2 l/s (7.47%, *P*=0.035), and maximum voluntary ventilation (8.58%, *P*=0.023) values were observed among aerobic athletes.

**Conclusion:**

Aerobic and anaerobic athletes had no statistically significant difference in forced vital capacity and forced expiratory volume in the first second; however, other spirometry parameters were comparatively better in aerobic athletes.

## Introduction

Highlights

- We aim to study the spirometry parameters between aerobic and anaerobic athletes.
- There was no significant difference in forced vital capacity, forced expiratory volume in 1 second, and forced expiratory volume in 1 second / forced vital capacity.
- The aerobic athletes had a significantly higher forced expiratory flow 25–75, forced expiratory flow 0.2–1.2, and maximum voluntary ventilation.
- This could be an adaptation to greater and prolonged ventilatory demand.

Regular physical exercise is beneficial for the human body with a positive effect on the respiratory system^1^. According to the WHO fact sheet, physical inactivity is the fourth main cause of mortality, and adults more than 18 years are advised to be involved in at least 150 min of moderate-intensity physical activity per week since physical activity has a positive impact on cardiorespiratory fitness^2^.

Athletes have better pulmonary function tests due to increased stress on the respiratory system during exercise to meet the metabolic demands during physical activity^3^. Exercise also helps to strengthen the muscles of respiration, thereby improving pulmonary functions, as supported by a comparative study between athletes and non-athletes in Nepal^4^. Therefore, it is unsurprising that higher aerobic fitness levels are associated with enhanced lung function^5^. The intensity and severity of sports involved by the athletes determine the extent of inspiratory muscle strengthening and the resultant increase in lung functions^4^. So, based on the nature of the activity, the respiratory parameters may also differ among athletes involved in anaerobic and aerobic sports.

Anaerobic (power) sports are defined as those sports where achievement depends on explosive muscle power, whereas aerobic (endurance) sports are those sports with prolonged and intensive high dynamic, often associated with high-static exercise^6^. Exercise is good for pulmonary function and is well known globally; however, the difference in pulmonary function between aerobic and anaerobic sports is less explored. Thus, we aimed to study the difference in spirometry parameters among aerobic and anaerobic athletes.

## Methods

### Study design and setting

A cross-sectional study was carried out on 131 professional athletes of a sports training centre. A convenience sampling technique was used. The demographic details, vital parameters, and spirometric data were collected from the study participants from April to November 2022.

### Study participants

Athletes playing under the sports category of anaerobic (power) or aerobic (endurance) were the study participants. A name list of all athletes was taken from official data and they were categorized into skill-based, aerobic, anaerobic, and mixed categories of sports. The sports included under the anaerobic category were weightlifting, wrestling, judo, and boxing. The aerobic sports included ultramarathon, marathon, triathlon, kabaddi, mid-to long-distance running, mid-to-long-distance swimming, cycling, and rafting.

Athletes trained for specific sport activity for more than six months were included in the study. Participation in at least one national or international competition was considered adequate training.

Athletes with any symptoms of cough/fever/chest pain on the day of the test, physical injury, a non-training period of more than 6 months, and those on annual leave during the study time were excluded. Athletes involved in sports other than aerobic or anaerobic like mixed sports or skill-based sports were also excluded.

In aerobic sports, there were 65 athletes present during the data collection period after inclusion and exclusion criteria, who were all enroled in the study. An equal number of athletes (*n*=66) in anaerobic sports were also chosen.

### Study instrument

A self-administered questionnaire containing 19 items was used. It contained seven items for demographic details and history, four items for clinical examination, and eight items for spirometry tests.

All the subjects reported to the place of testing after overnight fasting and refraining from any form of exercise for at least 3 h before the test. They were instructed not to smoke, consume alcohol, drink caffeinated beverages or take any medications containing theophylline or use beta-2 agonist inhalers before the test. Each athlete underwent anthropometric assessments, without shoes and minimal clothing. Body mass (kg) and stature were measured using the portable stadiometer. A digital fingertip pulse oximeter was used to measure the pulse. A digital sphygmomanometer was used to measure blood pressure.

The spirometry test was done between 8 and 10 am in a medical inspection room at the training site. The test was carried out at the same time of the day using the same instruments and technique. Measurements were carried out under standard environmental conditions; at a comfortable temperature between 20 and 24°C, the atmospheric pressure of 760 mmHg, and relative atmospheric humidity of 30–60%. The temperature, humidity, and atmospheric pressure were continuously monitored. Spirometry was performed using the Clarity SpiroTech Spirometer, model no CMSP-01, manufactured by Clarity Medical Pvt. Ltd, Punjab, India. The spirometry was performed following the ATS/ERS recommendations^7^. The calibration of the device was done as per the ATS/ERS recommendations, and also the maximum voluntary ventilation (MVV) was measured using the 12-s method as per the ATS/ERS recommendations.

They were made familiar with the machine and the objective of the study. The readings were recorded after sufficient practice. At least three acceptable manoeuvres were required for each subject, and the best of the three values was recorded. The highest values for forced vital capacity (FVC) and forced expiratory volume in 1 second (FEV1) were taken independently from the three curves. A *P* value less than 0.05 is considered statistically significant.

### Statistical analysis

The data were collected in the proforma form, entered into Microsoft Excel and analyzed using Statistical Package for Social Sciences (SPSS) version 16. The age, height, weight, resting pulse and blood pressure of the study participants were expressed in terms of mean (SD). An unpaired *t*-test was used to demonstrate the spirometry performance between aerobic and anaerobic groups. ANOVA test was used for evaluating spirometry performance among different sports activities.

### Ethical consideration

The study was approved by the Institution Review Committee (IRC) of the institution (reference number 612) in April 2022. Written informed consent was taken from each participant. The confidentiality of the study participants was ensured.

The manuscript has been reported in line with the STROCSS criteria^8^.

## Results

A total of 131 subjects agreed to participate in the study. The mean age of all participants was 27.34±5 years (range 19–40). The majority of the participants were male (*n*=104). All the participants were presently active professional athletes whose training duration ranged from 1–24 years (aerobic) to 1–20 years (anaerobic).

We classified the athletes into two groups according to the predominant character of sports they were associated with: the aerobic group (*n*=65) which included cycling, kabaddi, marathon, rafting, mid to long long-distance running, and swimming, triathlon, ultramarathon, and the anaerobic group (*n*=66) which included boxing, judo, weightlifting, and wrestling. There were 21 smokers in each group. Consumption of alcohol was found to be in 30 subjects in the aerobic group and 43 in the anaerobic group. The mean resting pulse rate and blood pressure were similar in both groups (Table 1).

**Table 1:** Demographic details and clinical examination findings among the respondents (*N*=131)

Parameter	Aerobic (*n*=65)	Anaerobic (*n*=66)
Mean age (years)	28.46±5.22	26.20±4.43
Sex (males: females)	56:9	48:18
Mean height of participants (m)	1.67±0.06	1.67±0.08
Mean weight of participants (kg)	60.12±7.77	66.83±14.49
Mean resting pulse rate (bpm)	69.2±8.53	68.03±9.06
Mean blood pressure (mmHg)	111.29/73.29±8.42/6.62	112.27/75.30±10.49/7.28

The spirometry performance was measured in terms of FVC, FEV1, FEV1/FVC, peak expiratory flow rate, Forced expiratory flow (FEF), lung age, and MVV. The difference in FEF 25–75 (13.42%, *P*=0.001), FEF 0.2–1.2 (7.47%, *P*=0.035), and MVV (8.58%, *P*=0.023) values among the aerobic and anaerobic groups were statistically significant (Table 2).

**Table 2:** Spirometry performance between aerobic and anaerobic groups (unpaired *t*-test)

Spirometry	Aerobic	Anaerobic	% difference (of anaerobic)	*P*
FVC (l)	4.24±0.76	4.19±0.85	1.19%	0.726
FEV1 (l)	3.68±0.64	3.57±0.61	3.08%	0.315
FEV1/FVC%	87.56±10.55	85.96±6.99	1.86%	0.308
PEFR (l/sec)	9.63±1.77	9.12±1.81	5.59%	0.102
FEF 25–75 (l/sec)	5.41±1.14	4.77±1.01	13.42%	0.001
FEF.2–1.2 (l/sec)	8.20±1.52	7.63±1.56	7.47%	0.035
Lung age (years)	19.12±9.26	18.62±9.63	2.69%	0.762
MVV (l/min)	174.19±35.97	160.42±32.24	8.58%	0.023

FEF, forced expiratory flow; FEV1, forced expiratory volume in 1 second; FVC, forced vital capacity; MVV, maximum voluntary ventilation; PEFR, Peak expiratory flow rate.

On performing the ANOVA test for sports performance among different sports activities, the measured parameters were not statistically significant (Table 3).

**Table 3:** Spirometry performance among different sports activities (ANOVA test)

Parameter	FVC	FEV1	FEV1/FVC%	PEFR	FEF 25–75	FEF.2–1.2	Lung Age	MVV
*P* value	0.343	0.418	0.681	0.794	0.055	0.471	0.356	0.112

FEF, forced expiratory flow; FEV1, forced expiratory volume in 1 second; FVC, forced vital capacity; MVV, maximum voluntary ventilation; PEFR, Peak expiratory flow rate.

## Discussion

Our study demonstrated that there were no statistically significant differences in commonly used pulmonary function parameters like FVC (1.19%, *P*=0.726), FEV1 (3.08%, *P*=0.315), and FEV1/FVC (1.86%, *P*=0.308) between aerobic and anaerobic athletes. However, the athletes involved in aerobic sports had a significantly higher difference in FEF 25–75 (13.42%, *P*=0.001), FEF 0.2–1.2 (7.47%, *P*=0.035), and MVV (8.58%, *P*=0.023) values than the athletes involved in anaerobic sports. FEF 25–75 indicates small airway obstruction while FEF 0.2–1.2 represents large airways. MVV is directly related to Breathing Reserve (BR), calculated as the difference between MVV and minute ventilation. These parameters are reduced in cases of primary lung diseases affecting ventilation, as in cases of obstructive lung diseases. Relatively higher MVV (and BR by corollary) in aerobically trained athletes compared to anaerobic athletes reflects better toleration to primary ventilatory lung diseases as compared with anaerobically trained athletes^9^. The greater pulmonary function in aerobic sports athletes could be explained by a greater demand for gas exchange and ventilation during exercise than in anaerobic athletes^10^.

Our findings were similar to a study from Serbia that aimed to compare the effects of sports on pulmonary functions, which showed no statistically significant difference in FVC, FEV1, FEV1/FVC, and peak expiratory flow rate between aerobic and anaerobic athletes^11^. Also, a cross-sectional study from Australia showed that there was no significant difference between strength-trained (anaerobic) and endurance-trained groups (aerobic) in terms of FVC, FEV1, and FEV1/FVC^3^. A randomized controlled trial from India comparing the effects of traditional aerobic exercise and sprint interval training on pulmonary function tests also demonstrated no significant difference in improvement in FVC (*P*=0.09) between the two groups^12^. However, a cross-sectional study from Serbia showed that the endurance group (aerobic) had significantly higher (*P*<0.01) FVC and FEV1, although no difference was seen in FEV1/FVC ratio compared to power athletes (anaerobic)^10^. Our study findings are not in line with a cross-sectional study, where endurance groups had significantly higher respiratory parameters like VC and FVC, and a significantly lower FEV1/VC than power groups^13^.

Endurance-trained (aerobic) groups had significantly greater MVV in studies from Australia (11.3%, *P*=0.02)^3^, and Serbia (*P*<0.05)^10,13^, findings similar to our study. The better spirometry performance among aerobic athletes could be due to the greater strength of respiratory muscles and a physiologic adaptation to a greater and prolonged ventilatory demand for aerobic exercise compared with anaerobic exercise^3,10^. However, in contrast to our study, there was no significant difference in improvement in MVV (*P*=0.29) between the aerobic and sprint interval training groups^12^.

There are a few limitations of our study as some factors like body fat percentage, muscle mass, fat-free mass, and waist-hip ratio that might affect pulmonary function^14^ were not considered in our study. Likewise, lung diffusion capacity and maximal oxygen uptake were not measured in our study. All athletes in the sports training centre are involved in the common basic training which includes a combination of aerobic and anaerobic exercises; the effect of this training was not measured in the research. Additionally, the details of the training in each group were not taken into account as it was presumed that they undergo scheduled training for that sport discipline under professional coaches.

## Conclusion

Our study showed that aerobic and anaerobic athletes had no statistically significant difference in FVC and FEV1. Other spirometry parameters like FEF 25–75, FEF 0.2–1.2, and MVV were significantly better in aerobic athletes. However, FVC and FEV1 are more widely used to measure lung volume, so involvement in any of these sports activities could benefit these spirometric parameters and respiratory health.

## Ethical approval

The study was approved by the Institutional Review Committee (IRC) of the Nepalese Army Institute of Health Sciences (NAIHS) with reference number 612 in April 2022. Written informed consent was taken from each participant. The confidentiality of the study participants was ensured.

## Consent

All the participants were informed about the study and its objectives during the time of data collection. The consent form was incorporated into the proforma. Written informed consent was taken.

## Source of funding

None.

## Author contribution

Y.S.R. and B.T.: literature review, conceptualization, methodology, data collection, formal analysis, writing—original draft, review and edit. L.B. and M.K.: literature review, methodology, data collection, writing—original draft, review and edit, supervision. S.R.L.R.: literature review, conceptualization, methodology, data collection, writing—original draft, review and edit. All the authors approved the final version of the manuscript.

## Conflicts of interest disclosure

No conflicts of interest.

## Research registration unique identifying number (UIN)

1. Name of the registry: research registry.
2. Unique Identifying number or registration ID: 8687.
3. Hyperlink to your specific registration (must be publicly accessible and will be checked): researchregistry.

## Guarantor

Dr. Yesha Shree Rajaure.

## Provenance and peer review

Not commissioned, externally peer-reviewed.

## Acknowledgements

The authors are thankful to all the study participants.
